# Latanoprost could exacerbate the progression of presbyopia

**DOI:** 10.1371/journal.pone.0211631

**Published:** 2019-01-31

**Authors:** Masahiko Ayaki, Yukari Tsuneyoshi, Kenya Yuki, Kazuo Tsubota, Kazuno Negishi

**Affiliations:** 1 Department of Ophthalmology, Keio University School of Medicine, Tokyo, Japan; 2 Otake Clinic Moon View Eye Center, Yamato, Japan; Bascom Palmer Eye Institute, UNITED STATES

## Abstract

**Purpose:**

Prostaglandin analogues (PG) reduce intra-ocular pressure by enhancing uveoscleral flow at the ciliary body, which controls accommodation via the ciliary muscle. We investigated the effect of PG on accommodation and presbyopia progression in glaucoma patients.

**Methods:**

We conducted a clinic-based, retrospective, cross-sectional study. Inclusion criteria were bilateral phakic patients aged 40–69 years with best corrected visual acuity better than 20/30. Exclusion criteria were any disease affecting vision other than glaucoma and history of ocular surgery. Subjects with no prescription or vision-affecting disease served as controls (n = 260). The glaucoma patients were prescribed eye drops containing 0.005% latanoprost for more than six months (n = 23). We measured the binocular near add power at a distance of 30 cm in both groups and compared the results using Kaplan-Meier analysis.

**Results:**

The mean age (± SD) of the control subjects was 51.5 ± 5.2 years and 39% were male. Similarly, the glaucoma patients had a mean age of 51.0 ± 7.2 years and 39% were male. There were no significant differences in age, gender, intra-ocular pressure, spherical equivalent, astigmatism, or anisometropia between groups. Survival analysis indicated that the glaucoma patients in this study reached the endpoint (near add power of +3.00 D) significantly earlier than control patients (*P* = 0.0001; generalized Wilcoxon test).

**Conclusions:**

Exacerbation of presbyopia progression in glaucoma patients is a potential side effect of latanoprost eyedrops.

## Introduction

Presbyopia and glaucoma are typical age-related eye diseases. Presbyopia is the loss of accommodation, and its progression predominantly depends on progressive lens hardening and decreased ciliary muscle mobility. It is an inevitable and irreversible part of the normal aging process, and a huge economic burden worldwide [1–6]. Glaucoma patients tend to be elderly and their visual function may have declined because of visual field loss, cataract, and presbyopia.

Latanoprost, a prostaglandin (PG) F_2α_ analog, is a first-line topical medication currently used to treat glaucoma because of its marked ability to reduce intra-ocular pressure (IOP) [7]. Although severe complications are rare, side effects associated with PGs include ocular surface complications, pigmentation of the skin and iris, conjunctival injection, and deepening of the upper eyelid sulcus [8,9]. The IOP-reducing effects of PGs are driven by an enhanced uveoscleral pathway, which involves tissue remodeling in the extracellular matrix of the ciliary muscle and the sclera [10–12]. The ciliary muscle also plays a major role in accommodation by altering the anterior curvature of the crystalline lens. Romano and Lograno [13] compared the effects of bimatoprost with the commonly prescribed PGs, latonoprost and travoprost, on human ciliary muscle contraction, and agreed with previous studies [14–19] that PGs might induce pseudomyopia. In contrast, pilocarpine, brimonidine, alpha agonists, and anti-inflammatory agents reduce presbyopia [20–25].

The aim of this study was to investigate the effects of PGs on accommodation. We focused particularly on potential long-term effects, such as chronic contraction of the ciliary muscle, which may affect vision especially in middle- to older-aged glaucoma patients. We achieved this by measuring the near add power in glaucoma patients prescribed with a 0.005% latanoprost ophthalmic solution and by comparing the progression of presbyopia between these patients and age-matched controls.

## Methods

### Study design and participants

This study was a clinic-based, retrospective, cross-sectional study involving glaucoma cases and control subjects attending the Jiyugaoka Ekimae Eye Clinic (Tokyo, Japan) from April 2015 to March 2017. The Institutional Review Board and Ethics Committee of the Jiyugaoka Ekimae Eye Clinic approved this study and the methods were carried out in accordance with the Declaration of Helsinki. Informed consent was obtained from all participants.

### Inclusion and exclusion criteria

Glaucoma cases and controls aged 49 to 70 years with bilateral phakic eyes and best-corrected visual acuity better than 20/30 were included. Individuals were excluded based on the following criteria: diagnosed with an ophthalmological disease other than normal tension glaucoma (NTG) and primary open angle glaucoma (POAG) that could potentially compromise visual acuity or contribute to visual field loss, including secondary glaucoma and age-related macular degeneration; history of ocular surgery. In addition, glaucoma patients were excluded if they had best-corrected visual acuity of 20/30 or less in either eye, did not use 0.005% latanoprost, or had used glaucoma medication containing 0.005% latanoprost for less than six months. Control patients were excluded if using any type of eyedrop.

### Ophthalmological examinations and diagnosis of glaucoma

Glaucoma was diagnosed using a visual field test (Humphrey Visual Field Analyzer 30–2 standard program; Carl Zeiss, Jena, Germany) and optical coherence tomography (OCT; RC3000; Nidek, Gamagori, Japan), which was used to measure the thickness of the ganglion cell complex. Routine ophthalmological examinations were also performed. Subjects with POAG and NTG were screened for eligibility with a battery of ophthalmic examinations including slit-lamp biomicroscopy, funduscopy, gonioscopy, IOP measurements, and visual field analysis using the 30–2 Swedish Interactive Threshold Algorithm standard strategy (Carl Zeiss, Jena, Germany). POAG and NTG were diagnosed when the following three conditions were present: (1) glaucomatous optic cupping represented by notch formation, generalized enlargement of cupping, senile sclerotic disc or myopic disc, or nerve fiber layer defects, (2) reproducible typical glaucomatous visual field defects such as Bjerrum scotoma, nasal step, or paracentral scotoma compatible with optic disc appearance, and (3) open angle observed on gonioscopy or slit-lamp biomicroscopy.

Topical glaucoma medications were Xalatan (0.005% latanoprost; Pfizer, Tokyo, Japan) and Xalacom (fixed combination with 0.005% latanoprost and 0.5% timolol maleate; Pfizer, Tokyo, Japan). One of the authors (MA) examined all glaucoma patients and controls, and reviewed medical records to check patient compliance by confirming the frequency and duration of visits, and the amount of prescribed latanoprost. Six months of latanoprost use was a sufficient period since previous studies [16,19] observed participants for 1 and 30 days to confirm significant effects.

Evaluation of control subjects included best-corrected visual acuity measurements, autorefractometry, slit-lamp biomicroscopy, funduscopy, and IOP measurements with a noncontact tonometer or Goldmann applanation tonometer. This group consisted mostly of individuals who visited the clinic for annual eye examinations, or who had an outer adnexal disease.

Binocular near add power was measured by a blinded examiner at a distance of 30 cm using a Bankoku near-acuity chart (Handaya Inc., Tokyo, Japan) or an automatic optometry system (AOS-700; Nidek, Gamagori, Japan). After determining the patient's distance refractive correction, the minimal additional power required to achieve near acuity better than 20/25 was measured in 0.25 D increments and was recorded as near add power.

### Statistical analysis

Patients’ demographics and ophthalmological parameters were compared using the *t* test and chi-square test. Near add power with an endpoint of +3.00 D was compared between glaucoma patients and age-matched controls using Kaplan-Meier survival analysis and the results analyzed using the generalized Wilcoxin test. Data are presented as the mean ± standard deviation (SD) or as percentages where appropriate. All analyses were performed using StatFlex (Atech, Osaka, Japan), with *P* < 0.05 considered significant.

## Results

**Fig 1:**
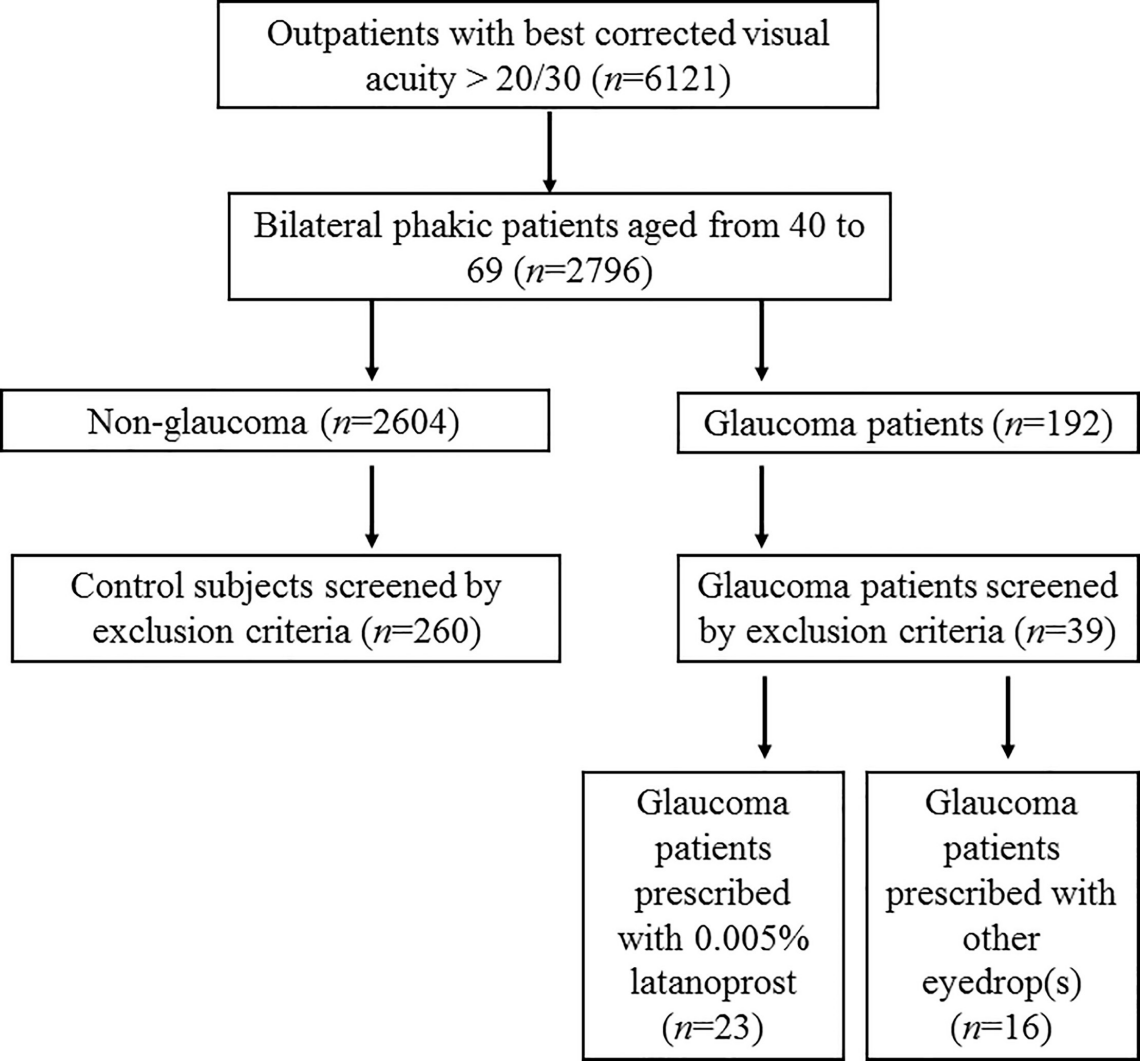
Flow diagram of patients’ enrolment and inclusion process.

Out of 6121 patients who visited the study institute during the study period, 260 control subjects and 23 glaucoma patients were finally analyzed following the stated inclusion and exclusion criteria (Fig 1). Xalatan was prescribed to 17 glaucoma patients and Xalacom was prescribed to 6 patients. Of those patients receiving Xalatan, four used hyaluronate, one used diquafosol, and one used rebamipide to alleviate dry eye. In addition, three patients used cyanocobalamin, levocabastine, and fluorometholone eyedrops, respectively. One patient prescribed Xalacom used hyaluronate. There were no significant differences in age, gender, IOP, spherical equivalent, astigmatism, or anisometropia between the groups (Table 1).

**Table 1:** Patient demographics and ophthalmological parameters.

	Control	Glaucoma	*P* value*
	n = 260	n = 23	
Age	51.0 ± 7.2	51.5 ± 5.2	0.339
Male (%)	39	39	0.835
Near add power	1.67 ± 0.99	1.83 ± 0.96	0.230
Spherical equivalent (diopter)	-3.75 ± 3.20	-5.09 ± 3.86	0.060
Astigmatic errors (diopter)	0.66 ± 0.71	0.53 ± 0.45	0.117
Anisometropia (diopter)	0.61 ± 0.66	0.54 ± 0.75	0.352
IOP (mmHg)	14.6 ± 3.0	14.0 ± 2.4	0.141
MD (dB)		-5.53 ± 3.98	
Optic Disc Cupping (%)		85.8 ± 9.0	
GCC (μm)		83.3 ± 15.5	

*P < 0.05, unpaired *t* test or chi-square test as appropriate.

Abbreviations: IOP, intra-ocular pressure; MD, mean deviation measured with the Humphrey Field Analyzer; GCC, thickness of the ganglion cell complex measured using optical coherence tomography.

**Fig 2:**
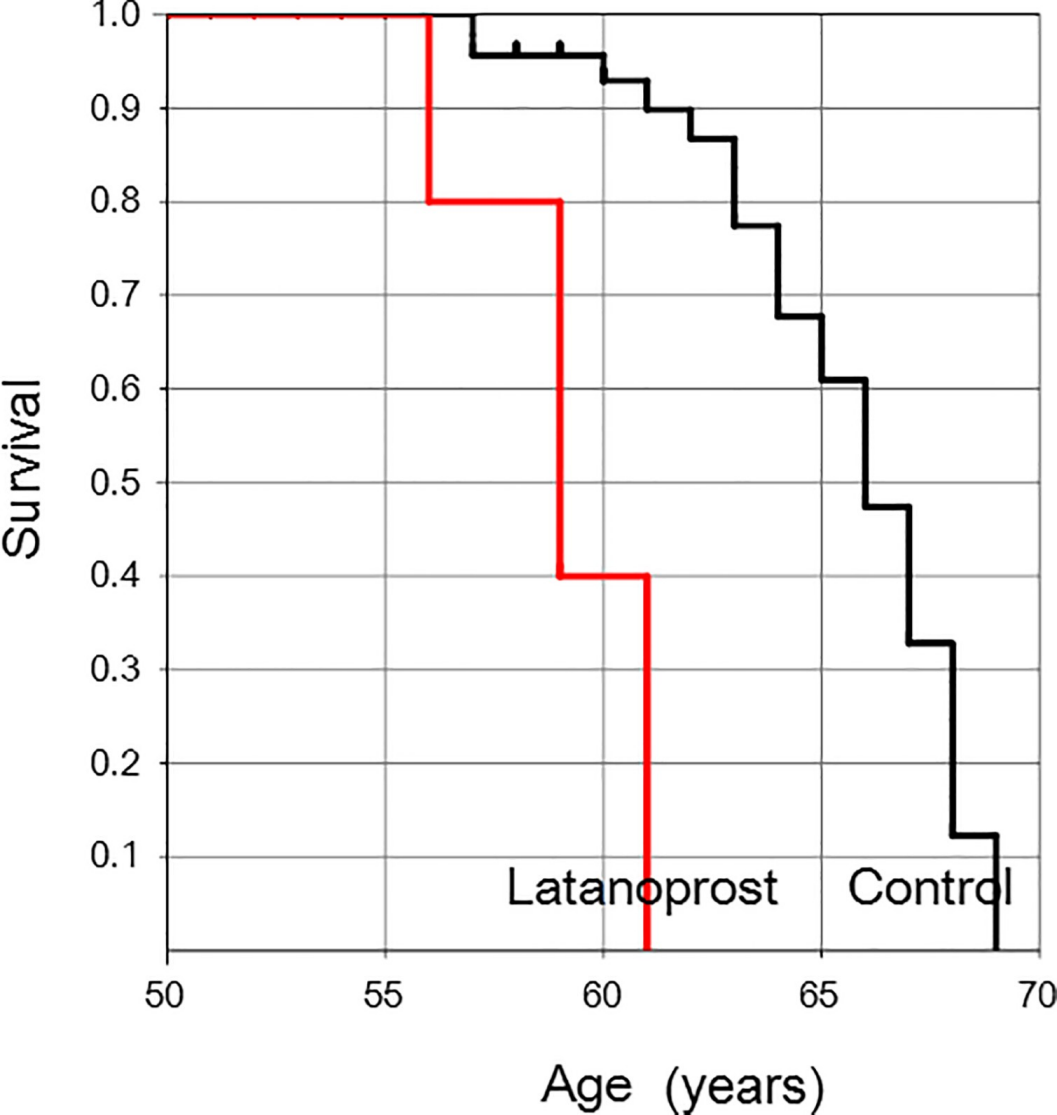
Kaplan-Meier survival plot showing the age at which individuals in the glaucoma and control groups reached the near addition endpoint of +3.00 D. Glaucoma patients reached the endpoint of +3.00 D significantly earlier than subjects in the control group (*P* = 0.0001; generalized Wilcoxon test).

Kaplan-Meier survival curve analysis indicated that glaucoma patients reached the endpoint (+3.00 D) significantly earlier than controls (*P* = 0.0001; generalized Wilcoxon test) (Fig 2).

## Discussion

The present study revealed that progression of presbyopia, as indicated by near add power, was accelerated in glaucoma patients with a history of latanoprost eyedrop use compared with healthy subjects with no history of eye disease or eyedrop use. Previous studies [16,19] indicated that latanoprost could reduce accommodation in young subjects after a single instillation and one month of use, and this study is the first study to be conducted in middle- to older aged patients. Although the contractility of the isolated ciliary muscle did not diminish with age, stiffened posterior ciliary muscle attachments might still play a possible role in restricting muscle and lens mobility during accommodation [10]. We speculate that latanoprost induces weak pseudomyopia and contraction of the ciliary muscle that may be reversible in young subjects, but become irreversible in the elderly. Ciliary muscle contraction might also become persistent with long-term latanoprost use. Consequently, long-term use may cause the muscle to stiffen and result in the apparent progression of presbyopia. Participating glaucoma patients used eyedrops other than latanoprost, including timolol malate, fluoromethoron, cyanocobalamine, and lubricating eyedrops. This is a possible confounding factor since a previous study showed some effect on timolol on the flattening of lens and ciliary muscle contraction [26], although the effect was still less than that of PGs and the other eyedrops have no reported effects on accommodation.

Based on the results of the present study, physicians and patients should be aware that latanoprost may worsen presbyopia. Glaucoma is a highly prevalent geriatric ocular disorder and PG is currently the most commonly used medical treatment option. Presbyopia worsens quality of life [27,28], and a potential side effect of PGs may be the rapid progression of presbyopia. Future research in this area should explore the long-term effects of other PGs, such as travoprost, tafluprost, and bimatoprost, and other antiglaucoma drugs on accommodation.

Despite the promising findings, this study has some limitations. The number of patients studied was small and the duration was short. A larger prospective study is required to confirm and provide more conclusive results about the progression of presbyopia in glaucoma patients using PGs. Glaucoma subjects also tend to be more myopic and this presents a possible confounding factor to the onset of early presbyopia among these subjects. Characteristics including systemic comorbidities, smoking, and other possible factors should also be investigated as potential contributors to presbyopia progression. Morphological assessment using ultrasound biomicroscopy or anterior segment swept-source optical coherence tomography would contribute in determining changed to the ciliary body itself during PG treatment. Measuring aberrations, anterior chamber depth, lens thickness, lens capsule curvature, and axial length could also provide knowledge about the structural changes that take place in the globe during PG treatment, while objective accommodation measures might further our understanding of the effects of PG on accommodation. Subclinical visual dysfunction is possible in patients with glaucomatous optic neuropathy, and in this study the central threshold should ideally have been checked using the Humphrey Field Analyzer 10–2 program; however, this was not conducted because there was no difference in best-corrected visual acuity between the glaucoma and control groups.

## Supporting information

S1 Table The raw data of the subjects. (XLS)
